# Reviewing the Prospects of Sea Fennel (*Crithmum maritimum* L.) as Emerging Vegetable Crop

**DOI:** 10.3390/plants7040092

**Published:** 2018-10-27

**Authors:** Massimiliano Renna

**Affiliations:** Department of Agricultural and Environmental Science, University of Bari Aldo Moro, via Amendola 165/A, 70126 Bari, Italy; massimiliano.renna@uniba.it; Tel.: +39-080-544-3098

**Keywords:** biology, cultivation, ethnobotany, health effects, food use, phytochemistry, proximate composition, SWOT analysis

## Abstract

Sea fennel (*Crithmum maritimum* L.), a perennial halophyte typical of coastal habits, is well known for several food and non-food uses. This review presents both the characteristics and ethnobotany as well as the findings, technical advances and potential of sea fennel research with the aim to improve and disseminate knowledge regarding the value and potentials of this halophyte. Current knowledge suggest that sea fennel shows good potential as an emerging crop, being a refined food and also an interesting source of human health compounds and crop protection products. Moreover, sea fennel may be proposed as an alternative and sustainable cash crop also in the context of a saline agriculture regime. On the other hand, some aspects of sea fennel require further understanding; therefore, new research and development activities should be carried out before full commercial exploitation.

## 1. Introduction

**Figure 1:**
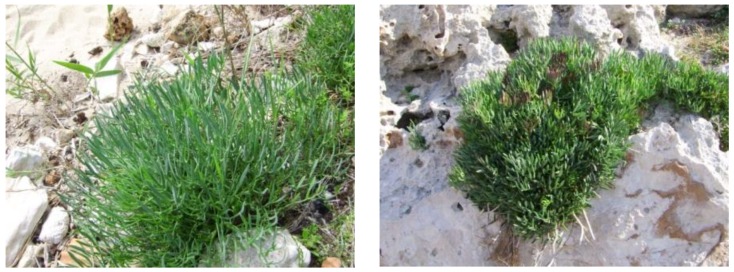
Plants of sea fennel on a sandy beach (**left**) and maritime rocks (**right**). Adapted from Renna et al. [2].

Sea fennel (*Crithmum maritimum* L.) is a perennial halophyte also known as samphire, crest marine, marine fennel and rock samphire which grows spontaneously on sandy beaches, maritime rocks, breakwaters and piers (Figure 1) of all the world’s coastlines, being particularly abundant along the coasts of Mediterranean countries [1].

In many parts of the world, sea fennel is used as a food ingredient for several traditional recipes. It is especially known for its good sensory traits [3], owed to its high essential oils content [3,4,5]. Furthermore, sea fennel is highly appreciated for both nutritional and medicinal uses, because the leaves contains several healthy compounds [2,6] and the seeds are rich in essential fatty acids [5]. At the same time, sea fennel is currently underutilized for commercial cultivation although it may be considered as a sustainable and promising crop, even if grown on saline soils [1,6].

Today, cultivation of vegetables and several other crops are facing many problems such as temperature increase, low availability of high quality water and soil salinization; these problems are attributable to climate change [7]. In this context, it is most important to have the possibility of proposing alternative crops, such as halophyte species, since they can better adapt under hard conditions, becoming suitable candidates as emerging cash crop and/or medicinal plants [8,9,10].

**Figure 2:**
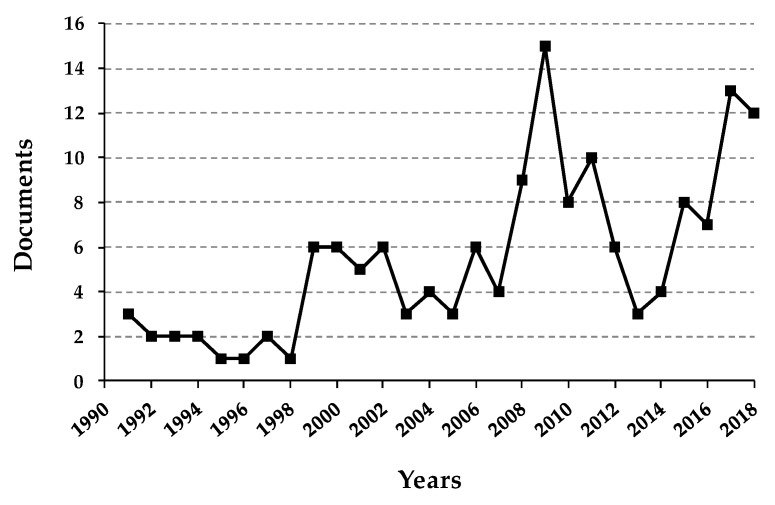
Documents regarding sea fennel published from 1991 to 2018. Documents by type: article (90.8%); conference paper (3.9%); review (3.3%); note (1.3%); book chapter (0.7%). Data retrieved from Scopus^®^ database by using “Crithmum” AND “maritimum” as key terms for searching.

Considering the growing scientific interest for this species (Figure 2), in the present review, both characteristics and ethnobotany will be presented as well as the findings, technical advances and potentials of sea fennel research with the aim to improve and disseminate knowledge regarding the value and potential of this halophyte.

## 2. Biology

**Figure 3:**
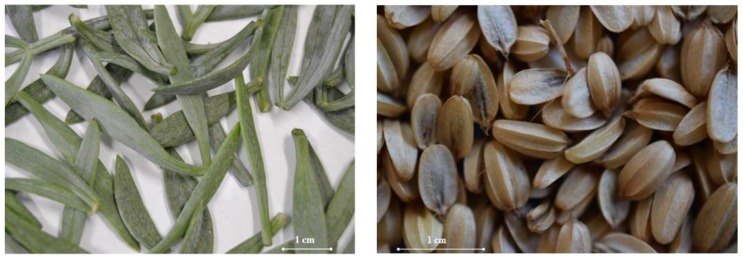
Leaves (**left**) and fruits (**right**) of sea fennel.

*Crithmum maritimum* L. is the only species of the genus *Crithmum* within the *Apiaceae* family. Sea fennel plants show many branches and succulent leaves (Figure 3) reaching a height up to 60 cm [11]. The blooming starts in June–September, while the fruits (Figure 3) do not begin to ripen before October–November [1].

*C. maritimum* L. produces a high amount of viable fruits that can germinate without any inconvenience, especially in distilled water [9]. Nonetheless, despite the fact that this halophyte usually grows near the seawater, seed germination is inhibited when salinities exceed 50 mM NaCl [9,10]. In the natural habitat, sea fennel fruits are always exposed to several ions such as Na^+^, Mg^2+^, Ca^2+^, Cl^−^, and SO_4_^2−^. Sodium salts adversely affect seeds germination firstly via the osmotic effect; it is not the case that a high germination recovery can be observed if seeds are transferred in distilled water [1]. Therefore, it is possible to speculate that in nature, although sea fennel seeds remain viable with high salinity conditions, the germination starts at the beginning of the spring, only after salt leaching which is due to the winter precipitations [1]. Another interesting aspect regarding sea fennel seed germination is the fact that it’s fruits can be considered a dispersal unit [11]. Both secretory envelope and spongy coat can protect sea fennel seeds from the damage due to potential Na+ and Cl− accumulation. Furthermore, the high floating capacity together with the germination recovery for explaining the long-distance seed dispersal and the consequent plants growth should also be considered [11].

Regarding the vegetative stage, some research reported that *C. maritimum* L. can be considered as a facultative halophyte [12,13]. By varying the salinity level during the vegetative stage, different eco-physiological responses can be observed. For example, Ben Hamed et al. [14] reported that plant growth was higher by using a nutrient solution with 50 mM NaCl as compared to a nutrient solution with 300 mM NaCl; in either case, no toxicity symptoms were reported. With a high salinity level, the antioxidant system activity was reduced [13,14], while the accumulation of toxic ions was higher [12]. Alternatively, the photosynthetic activity was maintained in the case of both low and high salinity levels [12]. Halophyte aptitude of sea fennel can be attributed to its ability to preserve tissue hydration and prevent oxidative stress [1,12,13].

## 3. Ethnobotanical Knowledge and Food Use

The term *Crithmum* is derived from Greek *krithe* (barley), probably due to the resemblance of fruits (Figure 3) to barleycorns; *maritimum* is due to its habitat, the sea [1]. In German, sea fennel is named *Meerfenchel* or *Seefenchel*; in French *fenouil marin*, *criste-marine*, *perce-pierr*, *passepierre*; in Italian *finocchio marino*, *cretamo*, *spaccasassi*, *bacicci*, *basiggia*, *erba di San Pietro*, *critama*; in Spanish *hinojo marino* or *perejil marino*; in Turkish *kaya koruğu* or *deniz rezenesi* [3,15].

In the past, sea fennel was used in folk medicine to prevent scurvy and for vermifuge and diuretic effects [1]. Today, sea fennel enjoys a good reputation as a traditional remedy in some Mediterranean regions. For example, in Spain, pickled leaves are eaten as a digestive and for its antiscorbutic and diuretic properties [16]. In Northern regions, the sea fennel decoction is used in folk medicine to take care of the urogenital apparatus and liver [17], while in Southern Italy, the same decoction is considered a useful remedy to treat whooping cough and cold [18]. At the same time, for inhabitants of central Italy, the leaf juice is traditionally used for its depurative, diuretic and carminative effect, whereas the fruit infusion is used for its stomachic, digestive and carminative properties [19].

In many countries, the fresh leaves of sea fennel are used to prepare soups, sauces and salads, or they are processed like capers in vinegar; canned sea fennel is registered in the “List of Traditional Agri-Food Product” by the Italian Ministry of Agriculture [2]. The “Rock Samphire Hash” is a traditional dish of the British Isles prepared by using sea fennel leaves mixed with pickled cucumbers and capers. According to Greek legends, the use of sea fennel as a food is lost in the mists of time considering that it was served to Theseus by Hekate [15].

**Figure 4:**
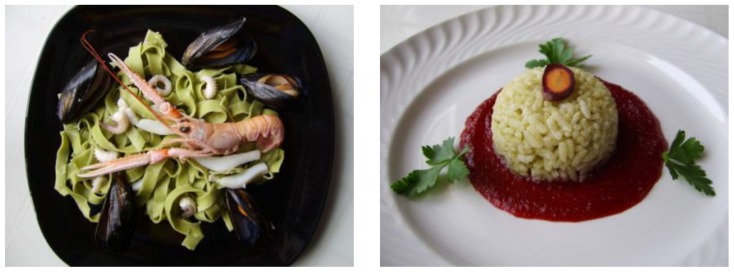
Green *tagliatelle* (pasta with sea fennel) in marinara style (**left**) and spiced “dome” (pilaf rice cooked with sea fennel) on puree of apple and purple carrot (**right**) [21].

Currently, there is a niche market which offers fresh-cut sea fennel for a broad number of uses in the culinary field [20]. Being an aromatic herb, sea fennel may be used not only as a fresh product but also as a dried herb [21]. Renna and Gonnella [21] reported the culinary use of the sea fennel as a new spice-colorant useful for several gastronomy products. According to these Authors, by using different types of dried sea fennel it is possible to obtain a broad range of applications in food; interesting flavor traits may primarily prevail in some cases, while a peculiar visual effect can be especially perceived in other cases. For example, in green *tagliatelle* (Figure 4), the main coloring effect of the freeze-dried sea fennel was highlighted, while in rice pilaf (Figure 4) with air-dried sea fennel the main spiced effect was reported as a primary trait [21].

With the aim to promote full exploitation of sea fennel, Renna et al. [2] proposed a new dehydrated sea fennel-based product by using several treatments such as air-drying, freeze-drying as well as microwave-drying or microwave-assisted air-drying. These Authors reported that all these treatments enabled a desirable water activity but with a significant reduction of essential oils and chlorophylls content. Both microwave-drying and freeze-drying premised to preserve colour parameters better than other treatments, while freeze-drying enabled the best colouring power [2]. Moreover, after the sensory analysis, the best scores were observed for microwave-dried, microwave-assisted air-dried and freeze-dried samples. In conclusion, results of this study suggest that both microwave-drying and freeze-drying are optimal for preserving qualitative traits of dried sea fennel for food use [2]. Finally, this study highlights the fact that dried sea fennel has impressive aromatic traits as well as an interesting colouring power similar to other food plant-derived colorants [2].

## 4. Phytochemistry and Composition

Proximate composition (content of water, lipid, protein, carbohydrates and ashes) of sea fennel is reported in Table 1.

Sea fennel is used for several culinary uses also for its fennel-like aromatic traits [2]. Thus, it is interesting to compare the proximate composition of sea fennel with that of common fennel (*Foeniculum vulgare* L.). Sea fennel shows a lower water content and a higher total lipid and protein content with respect to common fennel (Table 1). Total carbohydrate content seem to be the same for both species, nevertheless, see fennel shows a very low content of sugar and a relatively higher content of fiber with respect to common fennel (Table 1).

Regarding the content of some cations, Bianco et al. [22] found a higher content of sodium and calcium and a lower content of potassium and magnesium in wild sea fennel when compared to cultivated ones (Table 2).

This is because, in the marine coastal habitat, wild plants of sea fennel are more exposed to ions such as Na^+^ and Ca^2+^ with respect to K^+^. It is well know that potassium, although it is not a constituent of any plant structures or compounds, represents the element required in the highest amounts by plants for both biophysical and biochemical roles. Therefore, especially the higher amount of sodium in wild sea fennel could be a plant’s response to alleviating the negative effects of a non-optimal potassium availability level for vegetable tissues. This, considering the role of sodium in replacing potassium for both biochemical and physiological non-specific plant functions.

Beyond the macronutrient and cations content, sea fennel is rich in several chemical constituents. The essential oil of sea fennel contains several volatile compounds such as limonene, α-pinene, sabinene, p-cimene, β-terpinene, β-myrcene, thymol, γ-terpinene, carvacrol, p-cymol, β-ionone, dillapiole, anisaldehyde, β-caryophyllene, carvone and myristicine [1,23]. These volatile compounds are responsible for some sea fennel aromatic notes such as celery, common fennel and the peel of green citrus [21]. However, the essential oil composition of sea fennel can vary considerably based on the geographic origin of the plants; therefore, it could distinguish different chemotypes: aromatic monoterpenes-type, monoterpene hydrocarbons-type, phenylpropanoids-type and their intermediate forms [3,4,24,25,26,27,28,29,30,31,32]. Moreover, also other factors such as the life-cycle stage of the plant and year of collection, can affect the essential oil composition in sea fennel [3,4]. For example, Pateira et al. [4] found that the percentage of diallopil in sea fennel harvested from the same location and the same year was 42.1, 33.3 and 35.1% for vegetative, flowering and fruiting stage respectively. At the same time, these Authors found that the percentage of diallopile in sea fennel harvested during the vegetative stage was 42.1 and 14.5% in two different years of collection [4]. Apart from the composition, it is important to highlight that the amount of essential oils in sea fennel reaches about 0.8% in fruits and from 0.15 to 0.3% in leaves [1].

The oils extracted from *C. maritimum* L. leaves contain interesting amounts of fatty acids of the ω-3 and ω-6 series; their percentage (on dry weight basis—DW) reaches 2.02% for neutral lipids, 0.57% for the glycolipids and 0.26% for the phospholipids [33]. On the other hand, seeds contain about 44% (on DW) of oil, which is mainly constituted by oleic (78.6%), linoleic (15.4%) and palmitic acid (4.8%). This composition is similar to other oil types such as olive and canola, highlighting the high quality of oils extracted from sea fennel seeds [23].

Sea fennel leaves also contain a significant amount of other compounds such as ascorbic acid, carotenoids, tannins and flavonoids [15,33,34,35]. The content of vitamin C, flavonoids, tannins, total polyphenols and carotenoids in the aerial parts of sea fennel is reported in Table 3. It is interesting to highlight that this halophyte shows high phenolic contents compared to other crop species [36]. Chlorogenic acid and phenolic acids can be considered the most commonly detected phenolic compounds [37]. However, total phenols content of sea fennel can vary considerably based on the vegetation period.

**Figure 5:**
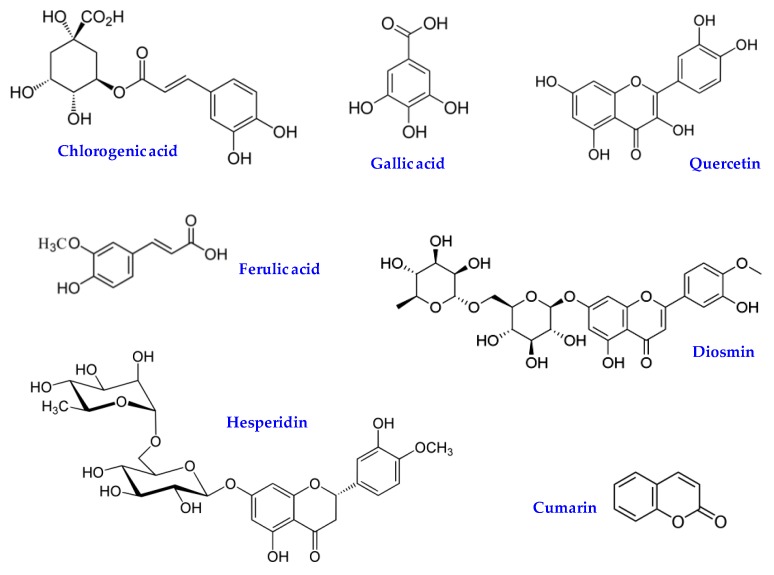
Chemical structure of some biologically active phenolic compounds found in sea fennel.

For example, Mekinić et al. [38] reported that during April, sea fennel extract contained the highest amount of phenolics and chlorogenic acid in respect to other months. The presence of diosmin and hesperidin was reported by Cornara et al. [39], while other Authors [37] reported high amounts of caffeic, gallic, rosmarinic, vanillic, and p-coumaric acids as well as a small content of *trans*-cinnamic and *trans*-2-hydroxycinnamic acids. A low amount of apigenin, rutin, quercetin-3-galactoside, catechin, epicatechin, epigallocatechin is also reported [37,40]. Meot-Duros et al. [40] reported the presence of falcarindiol in sea fennel leaves, while other Authors reported small amounts of some coumarins [23]. Figure 5 reports the chemical structure of some biologically active phenolic compounds found in sea fennel. Apart from phenolic compounds, other water-soluble compounds like sucrose, glucose and organic acids were reported [36].

Finally, another important aspect to highlight is the fact that sea fennel extract was proven to be highly sensitive to extraction techniques and extraction conditions. In a study aimed to evaluate the efficacy of two extraction techniques from *C. maritimum* L., Costa et al. [41] compared ultrasound-assisted extraction (UAE) and supercritical fluid extraction (SFE). The amount of total extract, contents of total phenolics and flavonoids and antioxidant capacity of the extracts were measured. The authors reported that by using UAE the amount of extracted antioxidants was approximately 10-fold higher compared to SFE. In particular, UAE was able to obtain the highest content of total phenols (23.44 mg Gallic Acid Equivalent g DW^−1^) and flavonoids (16.63 mg Quercetin Equivalent g DW^−1^). For UAE, best conditions were: temperature 50 °C, 20 min extraction time, 1:30 ratio solid:solvent and 40% (*v*/*v*) ethanol concentration. The best conditions for SFE were found at 50 °C and 300 bar pressures with 40% (*v*/*v*) ethanol concentration.

## 5. Biological Activity

Some biological activities of sea fennel are reported in Table 4.

Sea fennel contains interesting bioactive compounds that show antiradical, peroxidation inhibition and reducing power abilities [34,42]. In a study aimed to evaluate the antioxidant activity of different parts of sea fennel plant, Houta et al. [34] found that the methanolic extract of seeds displayed the highest DPPH· scavenging ability with the lowest IC50 value (406 μg mL^−1^) followed by leaves, flowers and stems (IC50 value of 500, 706 and 726 μg mL^−1^, respectively). According to some Authors [34,43], the strong antioxidant activity of the sea fennel extracts is due to the high content of phenolic compounds. However, it is important to highlight that both the content and composition of these compounds are highly influenced by the plant’s physiological stage [37]. The very high antiradical activity of sea fennel extracts reflects a high health potential in the context of functionality and antioxidant activity of food [32]. Apart from the soluble compounds, essential oils also show interesting antioxidant activity [44], although to a lesser extent [45]. For example, Mekinić et al. [45] found that the ferric reducing/antioxidant power (FRAP) of essential oils was of 22.0, 42.0 and 8.4 µmol Fe^2+^ L^−1^ respectively for flowers, stems and leaves, while the FRAP of ethanolic extracts was of 16,065.6, 3009.4 and 17,335.0 μmol Fe^2+^ L^−1^ respectively for flowers, stems and leaves. The same Authors found that the DPPH· scavenging ability (expressed as inhibition percentage of DPPH radical) of essential oils was of 2.8, 2.8 and 2.6% respectively for flowers, stems and leaves, while the the DPPH· scavenging ability of ethanolic extracts was of 61.0, 13.0 and 61.8% respectively for flowers, stems and leaves [45].

Sea fennel contains a significant amount of fatty acids within the ω-3 and ω-6 series; therefore, its use as a food could have beneficial effects toward the prevention of coronary heart diseases [33]. Mekinić et al. [45] reported a high vasodilatory activity when both plant extracts and essential oils were used. These Authors found that the maximal vasodilatory effect (expressed as the percentage decrease of the noradrenaline-precontracted rat aortic rings) for essential oils was 23.0%, while for ethanolic extract was 44.7% [45].

Moreover, sea fennel showed significant cytotoxic activity against some tumor cell lines such as mouse lymphocytic leukemia, lymphocytic leukemia and human myeloma without cytotoxic effects against some different mammalian cell lines [43] and normal human intestinal cells [40].

Another interesting aspect of sea fennel is its antimicrobial activity [34] being able to inhibit the growth of both Gram-positive and Gram-negative human pathogenic bacteria. Meot-Duros et al. [40] found that falcarindiol isolated from sea fennel leaves strongly inhibited the growth of *Micrococcus luteus* and *Bacillus cereus*, with a minimum inhibitory concentration (MIC) value of 50 µg mL^−1^.

Meot-Duros et al. [46] also reported a strong antimicrobial activity of sea fennel extract against *Salmonella arizonae*, *Erwinia carotovora*, *Pseudomonas fluorescens*, *P. marginalis* and *Candida albicans*.

In a study aimed to evaluate antitrypanosomal compounds in essential oils from the Apiaceae family, Ngahang Kamte et al. [47] reported that the essential oils from *C. maritimum* L. could be considered as a reservoir of substances to be used as leading compounds for the development of natural drugs for the treatment of Human African Trypanosomiasis.

Some authors [17,48] reported the veterinary use of sea fennel to integrate rabbit diet and stimulate milk production. In a study aimed to evaluate antifungal activity of sea fennel, essential oils against mushroom pathogen *Mycogone perniciosa* Mang., Glamoclija et al. [25] found better antifungal activities of essential oils than the commercial fungicide Prochloraz-Mn. It is also interesting to report that sea fennel could be used as a potential source of natural insecticides considering that essential oils showed a lethal effect against *Pheidole pallidulav* L. [27], *Sitophilus oryzae* L. and *Oryzaephilus surinamensis* L. [30]. Also, Pavela et al. [19] evaluated the insecticide effect of sea fennel, highlighting that essential oils rich in phenylpropanoids like dillapiole and myristicin show the best lethal effect against the larva of *Spodoptera littoralis* (Boisd.) and *Culex quinquefasciatus* Say, while essential oils rich in monoterpenes show only an inhibitory effect.

## 6. Cultivation

Although sea fennel can be considered as a promising crop, only little information is available in the literature regarding its growing techniques.

It is well known that factors such as temperature, salinity, and light conditions have a significant effect on the seed germination process and seedling production of sea fennel [49]. Marchioni-Ortau and Bocchieri [50] showed that the optimal conditions for seed germination of a Mediterranean population of sea fennel were a temperature of 20 °C and absence of light by using deionized water or at a very low salt concentration. It is important to highlight that the optimal conditions for germination of seed populations from the Atlantic coast seem to be very different from those reported for sea fennel populations from the Mediterranean basin [50]. In order to improve sea fennel seed germination for producing sea fennel seedlings, some interesting information is available in the literature. For example, Atia et al. [51] reported that the use of red light and nitrogen-based compounds can significantly promote and accelerate germination. At the same time, pretreatment of seeds with L-ascorbic acid or ethanol can improve the seed germination rate by 30% [52]. At any rate, seed priming may be carried out in order to accelerate seed germination as well as to obtain a better homogeneity as a consequence of an improved early seedling growth [53].

Some Authors [13,54] reported the production of sea fennel seedlings by in-vitro techniques without negatively affecting the performance of root and plant growth.

In order to assess salt response of sea fennel, Hamed et al. [12] cultivated this halophyte in pots filled with sand by using nutrient solutions at different NaCl concentrations (0, 50, 100, 150, 200, and 300 mM). Plants were harvested 13 weeks after transplanting while NaCl treatments started 8 weeks after transplanting. These Authors reported a biomass production of 1 and 13 g DW plant^−1^ by using a nutrient solution with 300 and 0 NaCl mM, respectively. Likewise, leaf area increased from 100 to more than 600 cm^2^ plant^−1^ with the same salinity levels, highlighting that sea fennel is moderately tolerant to salinity. Therefore, it is possible to consider *C. maritimun* L. as a facultative halophyte, since high growth does not necessary mean high salinity levels [12].

**Figure 6:**
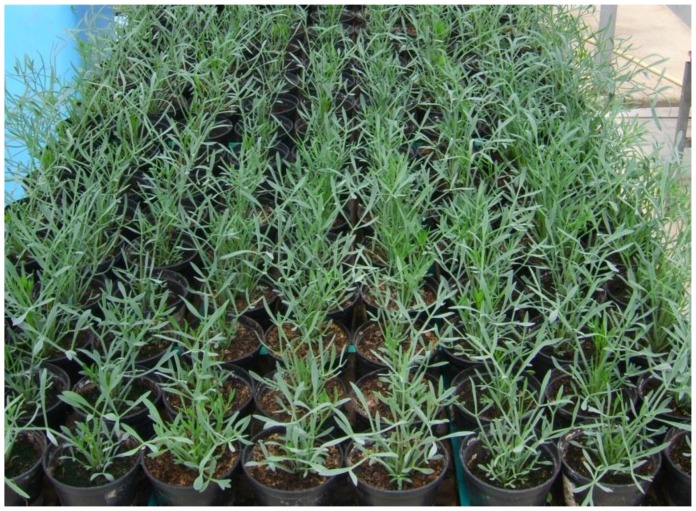
Potted sea fennel grown by using different posidonia compost-based substrates in comparison with peat [8].

Montesano et al. [6] evaluated the effect of posidonia compost-based substrates as a potential growing media for potted sea fennel cultivation (Figure 6). Following the harvest carried out 60 days after transplantation, these Authors found an average yield of about 5.4 g DW plant^−1^ without significant differences between various substrates, suggesting the possibility to replace peat substrates with renewable media without a negative effect on yield and quality. Furthermore, these Authors hypothesized that the halophyte aptitude of sea fennel can help to overcome the limitations posed by the high salinity of some compost-based growing media [6].

A lower sea fennel biomass production was found in other studies with values ranging from 2.1 to 3.0 g DW plant^−1^ [13,14]. Besides these studies, to the best of our knowledge, the literature lacks other information with regards to the cultivation of sea fennel.

In this regard, considering the differences in the mineral composition between wild and cultivated sea fennel plants [22], future research activities could be carried out with the aim to evaluate the effect of cultivation techniques on yield and quality of this facultative halophyte. For example, the use of different nutrient solutions for soilless production of sea fennel could be a useful way to affect the mineral composition in plant tissue and for biofortification purposes [55,56,57,58]. In this context, the implementation of a biofortification process in sea fennel cultivation could be carried out for improving its nutritional value. Being a facultative halophyte, it could also be useful assessing the effect of saline stress on minerals enrichment in sea fennel grown by soilless systems [59]. Finally, since data about open-field cultivation of sea fennel is not present in literature, the influence of soil composition and salinity on this cropping system should be evaluated to optimize yield and nutritional quality of this facultative halophyte.

## 7. Prospects

Apart from the nutritional traits and biological activity of sea fennel, several contributions are reported in literature regarding some culinary and folk uses of different sea fennel-based products (Table 5). Therefore, in order to evaluate whether sea fennel can be regarded as an emerging vegetable crop with a concrete chance to succeed, a SWOT analysis was performed. SWOT (Strengths, Weaknesses, Opportunities and Threats) analysis comprises the analysis of the strengths and weaknesses of a project, product, place or person and their relationship with the opportunities and threats of the surroundings. In short, it is a framework for identifying and analyzing the internal and external factors that can have an impact on the viability of a project, product, place or person. SWOT analysis could be considered as an important support tool for decision-making and is often used as a way to systematically analyze the internal and external environments of project, products and organizations. Table 6 reports the SWOT analysis regarding the exploitation of sea fennel as an alternative cash crop.

Regarding the strengths, it is important to first specify that sea fennel is a traditional food used for making various dishes and processed products. This halophyte shows interesting nutritional traits and taste; moreover, it is very appreciated as a functional food. Therefore, the development of a specific agri-food chain based on new processed sea fennel products could meet the demand for local functional food from growing markets. By contrast, the interesting traits of sea fennel as well as its use as a food are known only by researchers through scientific literature or by a small percentage of people in niche areas. Moreover, it must be highlighted that currently there is only a little market niche for sea fennel. These weaknesses require specific activities to be carried out in order to disseminate knowledge, promote potential uses and boost consumer demand. In this context, sea fennel exploitation could be carried out by a multi-disciplinary approach and integrated projects, such in the case of Slow Food Presidia [61].

Being a halophyte with great adaptation to salinization, sea fennel can be considered an alternative for both horticultural and industrial crops also in the case of low quality soils and/or the availability of irrigation water with high electrical conductivity. Moreover, this species can be proposed also as a potted plant by using sustainable growing media instead of peat-based substrates. Unfortunately, the cultivation of this halophyte is currently very limited, and therefore more knowledge and technical solutions are needed before any large-scale diffusion.

Thanks to its richness of health-promoting components, sea fennel seems to be a very promising candidate for both pharmaceutical and food industry in order to produce new functional products. At the same time, the richness in chemical compounds make this halophyte also interesting for industrial production of botanical insecticides and other crop protection products. At any rate, according to Petropoulos et al. [62], a multi-step approach could be need for hypothesizing a full commercialization of these new products. In this context, the evaluation of several sea fennel populations from different geographical areas should be firstly carried out in order to select the best chemotypes for specific uses. At the same time, it could be needed also an evaluation of the potential differences regarding bioactive compounds content of plants under commercial cultivation conditions with respect to wild plants or ones grown under experimental conditions. On the other hand, some threats may also arise due to the potential resistance of consumers and farmers regarding this species as functional food and/or new cash crop. This may require a few preventive activities including clinical trials for evaluating effects on health and a specific marketing project to achieve increased consumer acceptance [62]. In this context, the establishment of consortia between research institutes, business companies and governmental organizations aiming to carried out these research and development activities, could be a good opportunity.

## 8. Conclusions

Current knowledge suggests sea fennel shows good potential as an emerging crop, being a refined food and also an interesting source of human health compounds and crop protection products. Moreover, sea fennel may be an alternative for both horticultural and industrial crops when in the presence of low quality soils and/or availability of irrigation water with high electrical conductivity. Nevertheless, more knowledge is needed before any large-scale diffusion, including chemical composition depending on the geographical origin as well as the effect of commercial cultivation on yield and quality. Therefore, new research and development activities should be carried out in order to increase consumer and farmer acceptance of this species as a new functional food and/or alternative crop.

## Figures and Tables

**Table 1:** Proximate composition of sea fennel (*Crithmum maritimum* L.) and common fennel (*Foeniculum vulgare* L.).

Crop	Water	Total Lipid	Protein	Total Carbohydrate	Sugar, Total	Fiber, Total Dietary	Ashes
	g 100 g^−1^ FW						
**Sea fennel ^1^**	87.60	0.73	1.57	7.33	0.65	3.74	2.78
**Common fennel ^2^**	90.21	0.20	1.24	7.30	3.93	3.01	1.05

^1^ Data retrieved from Bianco et al. [22]; ^2^ Data retrieved from National Nutrient Database—United States Department of Agriculture.

**Table 2:** Average content of Na^+^, K^+^, Mg^2+^ and Ca^2+^ in wild and cultivated sea fennel (data retrieved from Bianco et al. [22]).

Sea Fennel Type	Na^+^	K^+^	Mg^2+^	Ca^2+^
	mg 100 g^−1^ FW			
**Wild**	291	335	28	310
**Cultivated**	168	588	41	250

**Table 3:** Content of vitamin C, flavonoids, tannins, total polyphenols and carotenoids in the aerial parts of sea fennel.

Compound	Value	References
**Vitamin C**	76.6 mg 100 g^−1^ FW	Franke [15]
**Flavonoids**	2.3 mg g^−1^ DW	Maleš et al. [35]
**Tannins**	6.8 mg g^−1^ DW	Maleš et al. [35]
**Total polyphenols**	2.3 mg g^−1^ DW	Maleš et al. [35]
**Carotenoids**	33.8 mg 100 g^−1^ DW	Guil-Guerrero et al. [33]

**Table 4:** Literature regarding biological activity of sea fennel.

Biological Activity	Product Type	References
Antibacterial	Essential oils	Loizzo et al. [42] Mekinić et al. [43]
Antibacterial	Plant extract	Meot-Duros et al. [39] Meot-Duros et al. [44] Loizzo et al. [42] Mekinić et al. [43] Houta et al. [35]
Antifungal	Essential oils	Glamoclija et al. [27]
Antifungal	Plant extract	Meot-Duros et al. [44] Houta et al. [35]
Antioxidant	Essential oils	Loizzo et al. [42] Mekinić et al. [43]
Antioxidant	Plant extract	Meot-Duros et al. [44] Loizzo et al. [42] Mekinić et al. [43] Houta et al. [35]
Cytotoxic against tumor cells	Plant extract	Meot-Duros et al. [39] Pereira et al. [41]
Insecticide	Essential oils	Tsoukatou et al. [28] Polatoglu et al. [32] Pavela et al. [21]
Milk production stimulant	Whole plant	Viegi et al. [45] Cornara et al. [19]
Vasodilator	Plant extract	Mekinić et al. [43]

**Table 5:** Literature regarding some uses of different sea fennel-based products.

Uses	Product Type	References
Agri-food	Pickled leaves	Renna et al. [2]
Aromatic herb	Potted plant	Montesano et al. [6]
Folk medicine	Fresh leaves	Atia et al. [1]
Folk medicine	Fruits infusion	Pavela et al. [19]
Folk medicine	Pickled leaves	Carrió and Vallès [16]
Folk medicine	Plant decotion	Cornara et al. [17] Savo et al. [18]
Folk medicine	Plant juice	Pavela et al. [19]
Food colorant	Dehydrated leaves	Renna et al. [2] Renna and Gonnella [21]
Gastronomy	Fresh-cut leaves	KopperCress [20]
Spice	Dehydrated leaves	Renna et al. [2] Renna and Gonnella [21] Giungato et al. [60]

**Table 6:** SWOT analysis related to the exploitation of sea fennel as an alternative cash crop.

**Strengths**	**Weaknesses**
● Traditional product	● Knowledge limited to local areas and researchers
● Good nutritional traits and taste	● Only niche market
● Richness in chemical compounds	● Limited cultivation
● Halophyte	
**Opportunities**	**Threats**
● Innovative products	● Resistance by consumers and farmers
● Slow Food Presidia	
● Consortia for R&D	
